# To control floating drug delivery system in a simulated gastric environment by adjusting the Shell layer formulation

**DOI:** 10.1186/s40824-021-00234-6

**Published:** 2021-10-09

**Authors:** Yu-Tung Hsu, Chen-Yu Kao, Ming-Hua Ho, Shiao-Pieng Lee

**Affiliations:** 1 Department of Chemical Engineering, National Taiwan University of Science and Technology, Taipei, 10617 Taiwan; 2 Graduate Institute of Biomedical Engineering, National Taiwan University of Science and Technology, Taipei, 10607 Taiwan; 3 Biomedical Engineering Research Center, National Defense Medical Center, Taipei, 11490 Taiwan; 4 R&D Center for Membrane Technology, National Taiwan University of Science and Technology, Taipei, 10617 Taiwan; 5 Division of Oral and Maxillofacial Surgery, Department of Dentistry, Tri-Service General Hospital, Taipei, 11490 Taiwan; 6 Department of Biomedical Engineering, National Defense Medical Center, Taipei, 11490 Taiwan

**Keywords:** Gastroretentive drug delivery, Core-shell particles, Floating beads, Chitosan, Xanthan gum, Anti-bacterial effect

## Abstract

**Background:**

Gastroretentive drug delivery system (GDDS) are novel systems that have been recently developed for treating stomach diseases. The key function of all GDDS systems is to control the retention time in the stomach. However, research into the bulk density or entanglement of polymers, especially regarding their effects on drug float and release times, is scarce.

**Methods:**

In this research, we prepared the floating core-shell beads carrying tetracycline. The ratio of chitosan and xanthan gum in the shell layer was changed to modify polymer compactness. Tetracycline was encapsulated in the alginate core.

**Results:**

Using scanning electron microscopy (SEM) techniques, we observed that the shell formulation did not change the bead morphology. The cross-sectional images showed that the beads were highly porous. The interaction between anionic xanthan gum and cationic chitosan made the shell layer dense, resisting to the mass transfer in the shell layer. Due to the high mass transfer resistance to water penetration, the longer float and delivery time were caused by the dense surface of the beads. The cell culture demonstrated that floating core-shell beads were biocompatible. Importantly, the beads with tetracycline showed a significant prolonged anti-bacterial effect.

**Conclusion:**

Research results proved that the floating and releasing progress of core-shell beads can be well controlled by adjusting the shell layer formulation that could promote the function of gastroretentive drugs.

## Introduction

Oral administration is the most common drug delivery method as it is multifunctional and convenient [1]. The size of drug carriers is usually adjusted to 1–2 mm, allowing the medicine in the stomach to pass through the pylorus and enter into the small intestine [2]. Advances in pharmaceutical techniques have enabled to release drugs in a specific position in vivo to lower toxicity, decrease side effects, and promote efficiency. Thus, the gastroretentive drug delivery system (GDDS) has been developed for treating stomach cancer, ulcer and infection. GDDS could keep drugs in stomach for a prolonged period to achieve a specific release, including several types: floating, mucoadhesive, expandable and rafting forming drug delivery systems.

The floating drug delivery system (FDDS) is also called a hydrodynamically balanced system (HBS). In FDDS, the drugs carriers float in gastric juice to ensure that drugs do not leave stomach shortly. It efficiently increases drug bioavailability by prolonging the release period. The variation of drug concentrations in blood is also decreased [3]. FDDS is a potential treatment for stomach and duodenum diseases. The density of FDDS carriers must be lower than those of the gastric juice and chymus. Therefore, medicines float and are slowly released in the stomach, compared with the convectional drug delivery methods. Most antibacterial agents have low minimum inhibitory concentration (MIC) to *Helicobacter Pylori* in vitro, but are not very effective for the eradication of infection caused by *Helicobacter Pylori* in vivo*.* The short residence time is the key problem [4]. Better stability and prolonged residence time allow more effective antibiotic penetration through the gastric mucus layer to suppress or eradicate *Helicobacter Pylori* in stomach [5, 6], which would be achieved by FDDS.

Currently, most FDDS would not float for 2–8 h; however, the drug float and release periods need to be prolonged. To obtain improved drug float and release times, previous research studies have widely investigated control of the drug carrier materials. Kawashima, Sato, Thanoo [7–10] et al. prepared hollow spheres for FDDS by using the emulsion-solvent diffusion method. These studies focused on controlling the carrier density by solvent diffusion. In particular, polymer porosity dominates the solvent diffusion rates that determining the carrier floating behaviors. Xu, Choi and El-Kamel et al. added gas-forming/generating agents in polymers to increase the porosity, and therefore, the floating properties [11–13]. Indeed, the porosity was influenced by the amount of gas-generating agents. According to prior researches, the gas generating agents and solvent diffusion would significantly affect the porosity in FDDS. In contrast, research into the bulk density or entanglement of polymers, especially regarding their effects on drug float and release times, is scarce.

In this research, we prepared the core-shell floating particles for GDDS. We used two polymers, chitosan and xanthan gum, as the shell layer, which are cationic and anionic, respectively. Chitosan and xanthan gum were widely used in previous researches for the drug delivery, so their biocompatibility and biodegradability have been well approved. Manca et al. controlled the ratios of chitosan and xanthan gum to adjust the surface potential of liposome with coating layer, which influenced the rheological properties of microparticles in aerosol performance [14]. In the study of Kulkarni et al., chitosan and xanthan gum were blended to produce dense particles. Their results supported that the chitosan/xanthan gum ratio influenced the mucoadhesive properties [15]. Fareez et al. prepared chitosan-coated alginate/xanthan gum beads. The surface properties of beads were determined by chitosan shell layer [16]. Although chitosan and xanthan gum were used in these studies, the core-shell and porous particles with shell layer controlled by chitosan and xanthan gum interactions have been never applied for the FDDS and GDDS system.

Adjusting the chitosan/xanthan gum (C/X) ratio enabled us to adjust the polymeric entanglement, and allowed us to control the shell layer properties and structures in floating beads. We studied how the shell layer affects the float, release and biocompatibility properties. Moreover, the efficient drug carriers with better duration in GDDS would be developed by controlling the shell layer properties.

## Materials and methods

### Materials

Alginic acid sodium salt (medium viscosity), xanthan gum (from *Xanthomonas campestris*), NaHCO_3_ (sodium bicarbonate powder), chitosan (low molecular weight), tetracycline (> 98.0%) were purchased from Sigma-Aldrich (St. Louis, MO). CaCl_2_ (calcium chloride) and HCl and acetic acid were purchased from J.T. Baker, Japan. Dulbecco’s modified eagle medium (DMEM), fetal bovine serum (FBS), penicillin, trypsin was purchased from Gibco Life Technologies (Thermo Fisher Scientific - TW). Kaighn’s modification of Ham’s F-12 medium was purchased from Manassas, VA, USA. MTT (3-(4,5-Dimethylthiazol-2-yl)-2,5-diphenyltetrazolium bromide) kit was purchased from Carlsbad, CA, USA. Distilled deionized (DI) water was used throughout the experiment.

### Preparation of floating beads containing tetracycline

Two solutions were prepared respectively for core-shell beads. One was the tetracycline-alginate aqueous solution for core, and the other was the chitosan/xanthan-gum (C/X) solution for shell. First, 50 mg tetracycline and 200 mg alginate were mixed in 10 ml DI water, and stirred for 2 h to obtain the tetracycline-alginate solution. 200 mg NaHCO3 was then added into alginate solution, and stirred at 500 rpm for 1 h.

On the other hand, the C/X solution was prepared by mixing chitosan solution and xanthan gum solution. The chitosan solution was fabricated by dissolving chitosan powders in 1 v/v% acetic acid aqueous solution, and xanthan gum solution was prepared by dissolving xanthan gum in DI water. After that, chitosan and xanthan gum solutions were mixed with different C/X ratios of 4:1, 4:2, 4:3, 4:4 and 1:4, where CaCl2 was also added as the concentration of 1 w/v%.

After finishing tetracycline-alginate solution for core and C/X solution for shell respectively, we extruded the tetracycline-alginate solution through a 26-gauge needle into the continuously stirred C/X solution. The C/X shell layer was then formed outside the alginate core, and the solution with floating beads was stabilized after continuously stirred for 15 min. After the beads were collected and washed with DI water, they were vacuum dried for 48 h and then stored in 4 °C until used. The exact formulations of core and shell layer were described in Table 1.

**Table 1:**
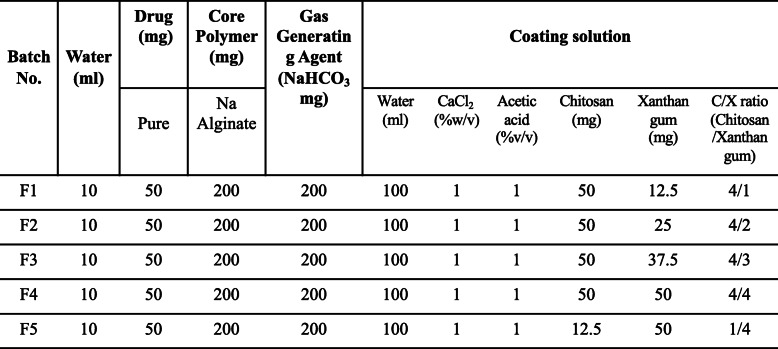
The formulations of core-shell floating beads with tetracycline

### FE-SEM (field emission scanning electron microscope) analysis

SEM (JEOL JSM-6390LV, Japan) images were taken to analyze the morphology of floating beads. The beads were dried at 4 °C. After that, the bead samples were spread and fixed on the metal plate with double-sided carbon tape, and a gold layer was then coated on the sample surface under vacuum using an auto-sputter coater for 1 min under argon atmosphere. The beads morphologies were then observed with SEM. With SEM images, the bead diameters were measured by Image J software.

### Swelling and floating test

The swelling percentage of beads was determined by measuring the extent of swelling of the polymer matrix in pH 2 aqueous solution. The weight of dried beads was recorded. After the immersion in pH 2 buffer for 1, 2, 4, 6, 8, 12 and 24 h, water on bead surfaces was removed by filter paper and the weight beads were measured again. The swelling percentage was calculated by using the following equation.

$$ \mathrm{Swelling}\ \mathrm{index}\ \left(\%\right)=\frac{\mathrm{Weight}\ \mathrm{of}\ \mathrm{beads}\ \mathrm{after}\ \mathrm{swelling}-\mathrm{Dry}\ \mathrm{weight}\ \mathrm{of}\ \mathrm{beads}}{\mathrm{Dry}\ \mathrm{weight}\ \mathrm{of}\ \mathrm{beads}}\times 100\% $$

For the floating analysis, twenty dried beads were kept in pH 2 buffer for 24 h with continuous shaking. After 1, 2, 4, 6, 8, 12 and 24 h, the floating percentage was calculated according to following equation:

$$ \mathrm{Floating}\ \mathrm{percentage}\ \left(\%\right)=\frac{N_f}{N_f+{N}_s}\times 100\% $$

N_f_: number of floating beads; Ns: number of settled beads

### In vitro release and encapsulation efficiency of tetracycline

The dry beads were immersed in pH 2 buffer in the dynamic gastric simulator [17]. After 2, 4, 6, 8, 12 and 24 h, 10 ml solution was taken out and analyzed by UV/VIS spectrophotometer at 266 nm, followed by refilling fresh 10 ml buffer.

In the analysis of encapsulation efficiency, beads were suspended in pH 2 buffer with continuous shaking at 37 °C for 24 h, where a part of encapsulated tetracycline was released. Then, the beads were completely broken by using ultrasonic shaker for 30 min, and the residual tetracycline would be completely released. After the solution was filtrated, UV/VIS spectrophotometer was applied to quantify tetracycline in buffer by deducting the absorbance at 266 nm, allowing the determination of encapsulation efficiency as following equation.

$$ \mathrm{Encapsulation}\ \mathrm{Efficiency}\ \left(\mathrm{EE}\%\right)=\frac{\mathrm{Actual}\ \mathrm{loaded}\ \mathrm{drug}}{\mathrm{Amount}\ \mathrm{of}\ \mathrm{drug}\ \mathrm{added}}\times 100\% $$

### Cytotoxicity test

MTT assay was applied to evaluate the cytotoxicity of core-shell beads. In this experiment, a stomach cell line, AGS, was seeded on a 24-well plate with the density of 5 × 10^3^ cells per well. AGS has been applied in the cytotoxicity of biomaterials for stomach in previous researches [18]. As the cell monolayer were cultured to confluence, they were exposed to fluid extracts. The extracts were obtained by placing the core-shell beads in culture medium (0.2 g core-shell particles in 1 ml medium) for 24 h at 37 °C. The C/X = 4:3 core-shell beads were applied in this research. Each fluid extract obtained was then applied to AGS monolayer, replacing the medium that had nourished the cells. The cells were then cultured with extracts for 1 day. After the cell culture, the metabolic activity of AGS was determined by MTT assay. The cells were incubated with 1 mg/mL of MTT for 4 h. Then, the MTT was removed and the formazan crystals were dissolved with dimethyl sulfoxide for 30 min. Finally, absorbance values were read at 570 nm by using an automatic microplate reader (ELx800; Bio-Tek Instruments, Winooski, VT, USA).

### Antibacterial testing

LB medium (from Creative Life Science Co., Ltd.) was applied for the culture of *E. coli* which was from Bioresources Conservation and Research Center (BCRC), Taiwan. The medium with cultured bacterial was added onto agar plate evenly, followed by an overnight culture. After that, core-shell beads were added onto the agar plate, and the antibacterial effects were observed at various time points.

### Statistical analysis

The one-way ANOVA was used to analyze the statistical significance of particle diameters, encapsulation efficiency, floating percentage, and cell viability. The two-way ANOVA was used to analyze the statistical significance of swell percentage.

## Results

Table 1 demonstrates the floating bead formulations. In this research, the mass ratio of chitosan to xanthan gum (C/X) in the shell layer was adjusted to 4:1, 4:2, 4:3, 4:4 and 1:4, where the amounts of salts (CaCl_2_ and NaHCO_3_) and alginate in core were fixed.

**Fig. 1:**
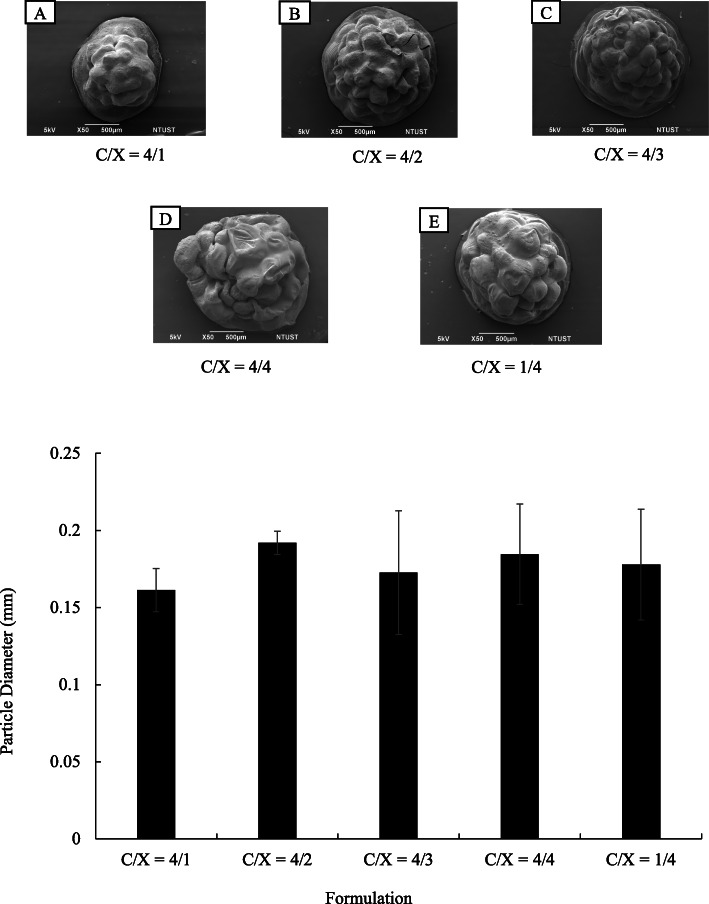
Morphologies of core-shell floating beads with various formulation in chitosan/xanthan-gum shell layer (*n* = 20). (a) SEM images of floating beads. The C/X ratios are 4:1 (A), 4:2 (B), 4:3 (C), 4:4. (D) and 1:4 (E). (b) Diameters of beads with various formulations in chitosan/xanthan-gum (C/X) shell layer (*p* > 0.1 form ANOVA test, *n* ≥ 10)

**Fig. 2:**
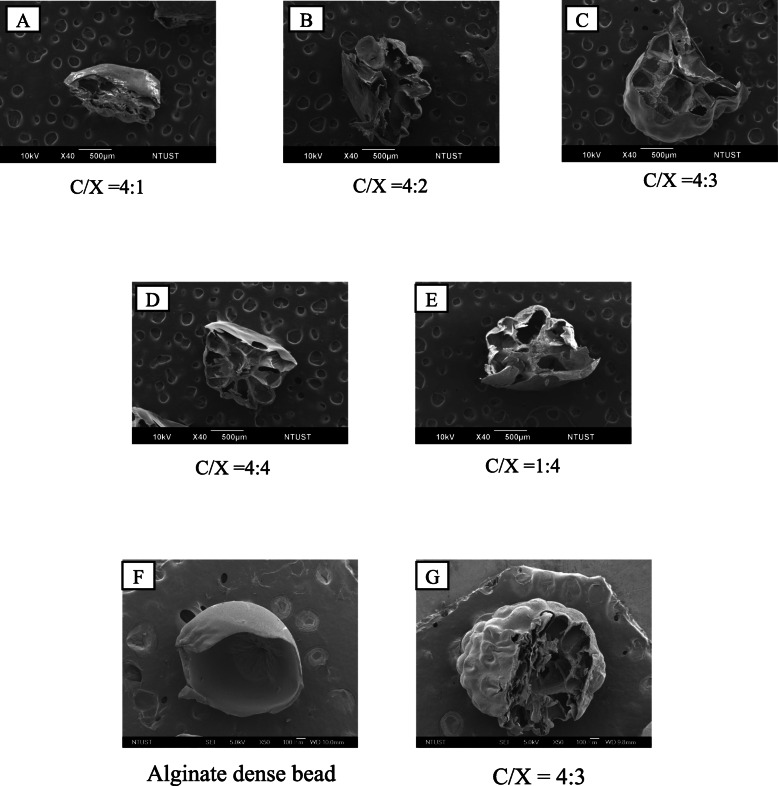
The cross-sectional SEM images of floating beads with various formulation in chitosan and xanthan gum shell layer. The C/X ratios are 4:1 (A), 4:2 (B), 4:3 (C), 4:4 (D) and 1:4 (E). The magnified images of alginate dense bead (F) and floating beads with C/X = 4:3 (G) were presented to identify the dense and porous structures in beads

The morphologies of core-shell particles were analyzed by using SEM as shown in Fig. 1(a). The beads were roughly spherical, with a diameter ranging from 1.5 to 2 mm. The particle diameters were analyzed using Image J and presented in Fig. 1(b). The formulation of the shell layer had a weak impact on the particle morphologies and diameters. The ANOVA analysis indicated *p* > 0.1 for the formulation effects on particle diameters, revealing the ratios of chitosan and xanthan gum did not change particle size significantly. The cross-sectional images of core-shell beads are presented in Fig. 2. Results show that the particles prepared in this research were highly porous. On the other hand, the surfaces of floating beads were highly dense. The whole particle was highly dense when only alginate was applied to prepare dense bead.

**Fig. 3:**
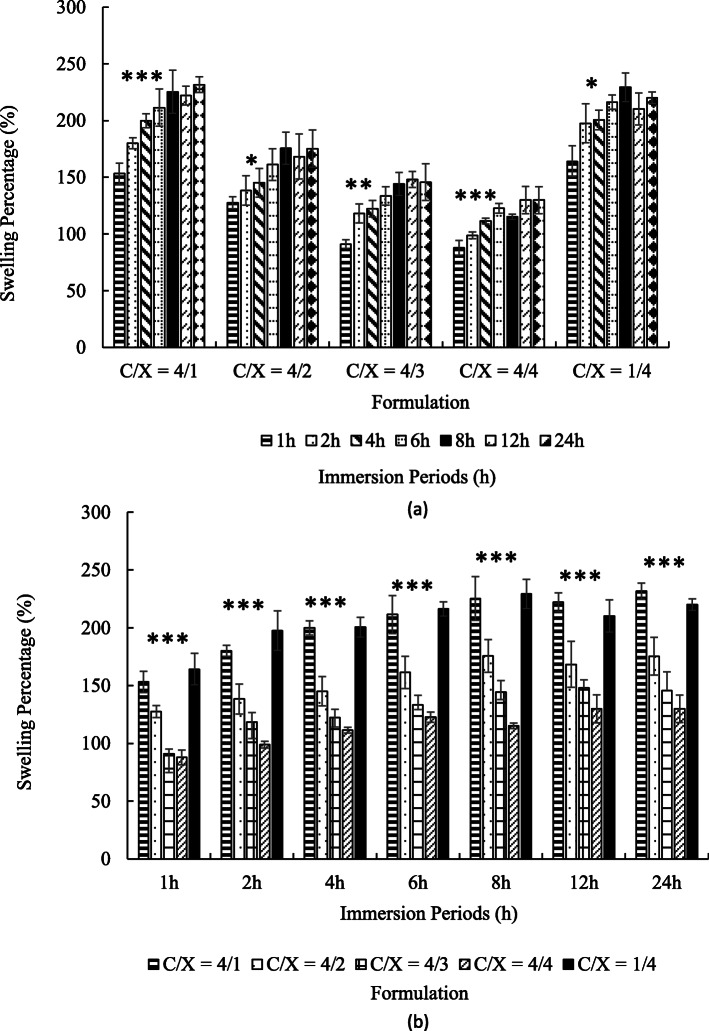
The swelling ratios of core-shell beads with various in chitosan/xanthan-gum (C/X) formulation in pH = 2 buffer (*n* ≥ 4). The times for immersion were 1, 2, 4, 6, 8, 12 and 24 h. The results were pooled in accordance with formulation (a) and with immersion period (b). In (a), the significance from ANOVA test from 1 h to 6 h with fixed formulation was marked by * (*p* < 0.05), ** (*p* < 0.01) and *** (*p* < 0.005) for indicated groups. In (b), the significance from ANOVA test for C/X ratio with fixed immersion time was marked by *** (*p <* 0.005)

Figure 3 revealed the swelling ratios of core-shell particles with different immersion periods at pH 2. As shown in Fig. 3(a), all kinds of particles were gradually swelled during first 6 h (*p* < 0.05 from ANOVA test) and reached a steady state over the next 2 h. The highest swelling ratios at steady state were 150–250%.

Figure 3(b) shows that the particles are swelled faster and steady-state swelling ratios increased when the amounts of chitosan were much higher or much lower than the amounts of xanthan gum, such as C/X = 4:1 and 1:4. In contrast, the swelling ratios were relatively low with C/X = 4:3 and 4:4. At all the given time point, C/X = 4/1 and 1/4 showed the highest swelling ratio, and the second high were C/X = 4/2. The lowest swelling ratio appeared in C/X = 4/3 and C/X = 4/4. All differences mentioned in this paragraph were significant according to t-test (*p* < 0.05).

**Fig. 4:**
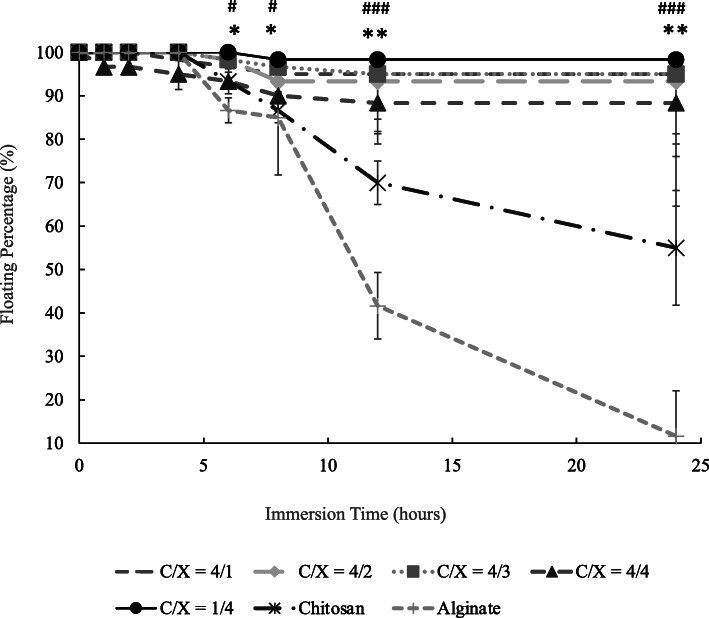
The floating percentage of core-shell beads with various chitosan/xanthan gum ratios, chitosan beads and alginate beads (*n* ≥ 3). The significant difference between core-shell beads with all the formulations and dense chitosan beads was indicated by * (*p <* 0.05) and ** (*p <* 0.01) from t-test at the same immersion time. The significant difference between core-shell beads with all the formulations and dense alginate beads was indicated by # (*p <* 0.05), ## (*p <* 0.01) and ### (*p <* 0.005) from t-test at the same immersion time (*n* ≥ 3). Chitosan and alginate beads were dense particles without core-shell structure. There is not significant differences between floating beads with different C/X (*p* > 0.15) from ANOVA test

Figure 4 presents the floating percentages of core-shell beads in the aqueous solution with pH 2 after immersion for different periods. Figure 4 shows that the floating percentage of chitosan and alginate beads respectively decreased to 55 and 11.6% after 24 h when there is no core-shell structure. Results in Fig. 4 also show that about 90 and 88% of core-shell particles would still keep their floating conditions after 8 and 24 h. The differences between core-shell beads and non-core-shell beads (dense chitosan and alginate beads) were statistically significant after the immersion for 6 h. On the contrary, the ANOVA test indicated that there is no significant difference caused by the ratios of chitosan and xanthan gum (*p* > 0.15).

**Fig. 5:**
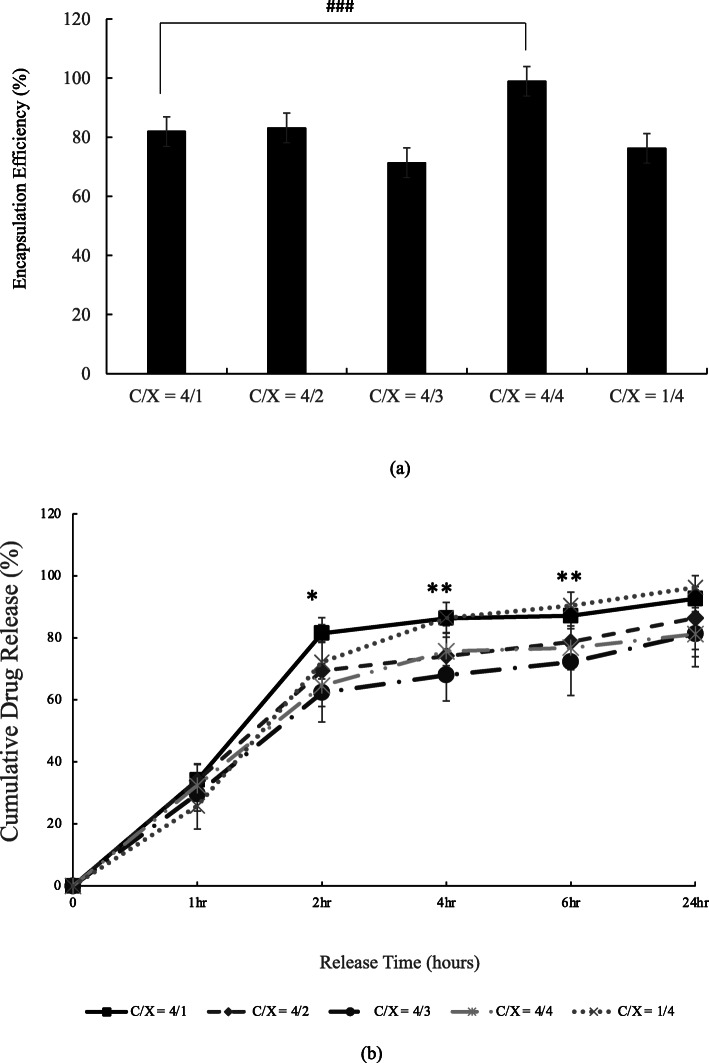
Encapsulation efficiency (a) and releasing profile (b) of tetracycline-alginate floating beads in pH = 2 buffer. In (a), the significant differences were indicated by ### (*p <* 0.005) from t-test (*n* ≥ 3). In (b), the significant differences were indicated by * (*p <* 0.1) and ** (*p <* 0.05) from ANOVA test with the same release time (*n* ≥ 4)

The encapsulation efficiency and releasing rate of tetracycline of floating beads are described in Fig. 5. There was significant difference in the formulation C/X = 4/1 and C/X = 4/4, as revealed in Fig. 5(a). The release profile in Fig. 5(b) was evaluated in dynamic conditions. The results support that the formulation of shell layer actually influence the releases of tetracycline (*p* < 0.1 and *p* < 0.05 from ANOVA test) at 2nd, 4th and 6th hr. That is, the particles released tetracycline faster when the amounts of chitosan are much higher or much lower than the amounts of xanthan gum, such as C/X = 4:1 and 1:4.

**Fig. 6:**
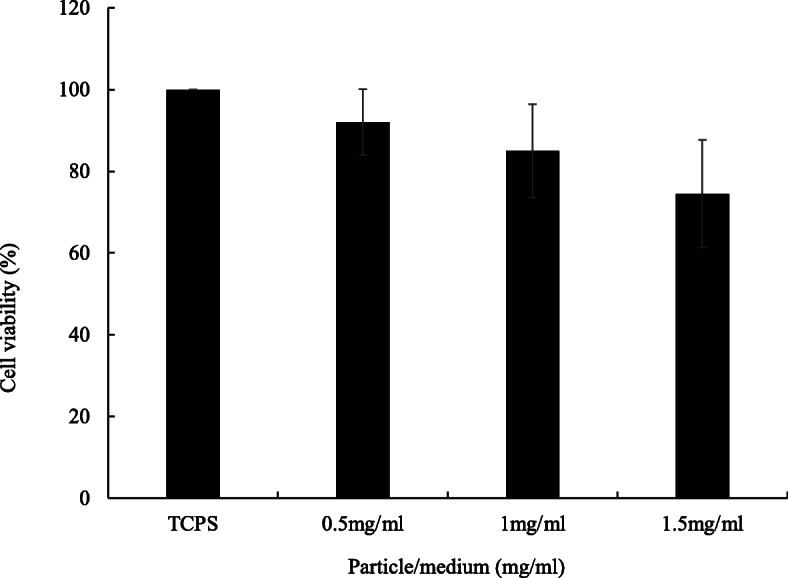
Biocompatibility of floating beads. The bead amounts in culture medium were 0.5, 1 and 1.5 mg/ml, respectively. Floating beads are core-shell beads with C/X = 4/3. TCPS was tissue culture polystyrene which was used as the controlled group. The culture period was 24 h. From ANOVA test, there was no statistical difference (*p* > 0.05 and *n* ≥ 4). From t test, all the core-shell bead groups were not significantly different from TCPS (*p >* 0.05 and *n* ≥ 4)

The biocompatibility of floating particles was analyzed by culturing AGS cell line. The C/X = 4:3 core-shell beads were applied because they showed the best floating and long-term release properties in this research. The results in Fig. 6 identify the good biocompatibility of floating beads when the bead concentration was as high as 1.5 mg/ml, which contained 149.03 μg/ml tetracycline, 112.9 times higher than the effective concentration of tetracycline for a 60-kg person [17].

**Fig. 7:**
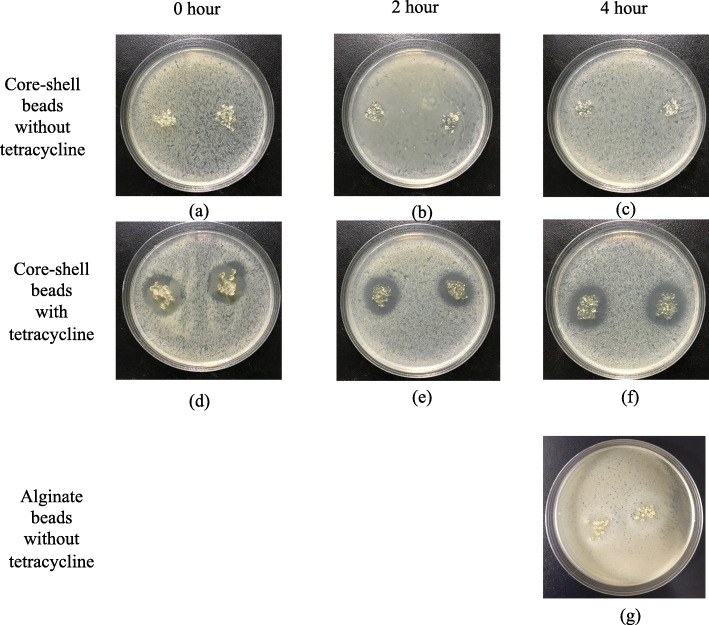
Antibacterial effects of floating beads with/without tetracycline for different immersion periods. (a), (b) and (c) are core-shell beads (C/X=4/3) without tetracycline, and (d), (e) and (f) are core-shell beads (C/X=4/3) with tetracycline. (g) is non-core-shell alginate beads without tetracycline. The immersion time before antibacterial test is 0 hour for (a) (d), 2 hours for (b) (e), and 4 hours for (c) (f) and (g). The beads in (d), (e) and (f) encapsulate 69.9 μg tetracycline in total

The anti-bacterial effects of floating beads are proved in Fig. 7. The obvious anti-bacterial rings are formed by applying beads with encapsulated tetracycline onto cultured *E. coli.* It was caused by the released tetracycline, and the core-shell beads without tetracycline did not result in any anti-bacterial ring (Fig. 7(a), (b), and (c)). To identify the duration of the floating particles, we immersed the particles in a pH 2-buffer for 2 and 4 h before conducting the anti-bacterial experiments. The results in Fig. 7(e) and (f) proved that the beads can efficiently suppress *E. coli* though there was a pre-release for 2 and 4 h. Compared with the beads without prerelease in Fig. 7(d), the anti-bacterial effect did not decay, as shown in in Fig. 7(e) and (f). Figure 7(g) presented the anti-bacterial effects of alginate beads without tetracycline. The alginate beads were immersed in a pH 2-buffer for 4 h before the test. The result shows that the alginate bead is not effective on *E. coli*.

## Discussion

The densities of chitosan and xanthan gum used in this research were approximately 0.3 and 1.5 g/cm^3^, respectively. The bulk densities of blended chitosan and xanthan gum were approximately 1.47 g/cm^3^ when the ratio was 1:1. After get mixed, chitosan and xanthan gum demonstrated high density compared with their original values. Thus, we assume that interactions between chitosan and xanthan gum increase the density of the two components. The anionic polymer chains of xanthan gum and protonated chitosan exert high intermolecular forces because of the electro-affinity. Polymer interactions make the shell layer dense. In previous researches, polyelectrolyte complexes (PECs) were prepared by blending chitosan and polyanions, such as carrageenan [19], alginate [20], poly (acrylic acid) (PAA) and poly (vinylpyrrolidone) (PVP) [21]. By varying the amounts of positive chitosan and negative polymers, the charge density would be changed, causing differences in the diffusion through PECs. The mass transfer properties were highly related to the polymeric electrostatic interactions, corresponded to our finding in this research.

According to SEM images, the bead surfaces are dense, which would prolong floating periods due to the resistance of mass transfer in water penetration. The dense skin layer could be help prolong the release time of encapsulated drugs. The C/X ratios did not significantly affect the porosity of core-shell beads.

The positive charges of chitosan and negative charges of xanthan gum would result in a strong polymer interaction. These interactions make the shell layer dense, forming a resistance of mass transfer in the shell layer. This hinders water penetration into particles, and therefore, the swelling ratio is low. This result in Fig. 3 revealed that the particle swelling can be tuned by controlling the electro-statistical properties of the shell layer. Besides, the high swelling ratios of core-shell beads show that all the beads developed in this research are very hydrophilic. The hydrophilic particles can provide good encapsulation efficiency and release profile due to the high affinity between the drugs and particles.

The retention periods of FDDS in the stomach reported in previous studies were about 2–4 h [22]. The floating time of core-shell particles was about 24 h, which was much longer than the above residual periods. It indicates that the dense shell layer developed in this research can prolong the floating time of drugs in the stomach.

When the amounts of chitosan are much higher or much lower than xanthan gum (C/X = 4:1 and 1:4), the release of tetracycline is also higher than those from C/X = 4:3 and 4:4. This is due to the strong interactions between positive chitosan and negative xanthan gum, which results in a dense shell layer. With the dense shell layer, the particle swelling is slow, and the tetracycline release is delayed due to the high resistance in mass transfer. The results supported that the prolonged release can be achieved by controlling the formulation of the dense layer in the core-shell floating beads.

Many researches supported that the ionic crosslinking, which was achieved by mixing chitosan and polyanions, was effective on the control release [23–25]. In this research, we adjusted the ratio between positive chitosan and negative xanthan gum, and the diffusion properties were thus controlled. The differences between this study and previous researches lies in the components diffusing through polycation/polyanion layer. The water diffusion through shell layer was influenced in this work to prolong the swelling and floating periods of core-shell porous beads. On the other hand, most previous researches focused on the controlled release of encapsulated drugs but not on the floating behaviors.

The floating beads are proved to be biocompatible, and can carry effective antibiotics when they were applied. The released tetracycline from core-shell beads present clear antibacterial effects. According to the statistical analysis, the C/X ratio significantly prolonged the swelling (*p* < 0.005 from ANOVA test) and drug release (*p* < 0.1 and *p* < 0.05 from ANOVA test). This supports that the core-shell beads developed in this research can continuously delivery antibiotics for a long period for a certain period.

## Conclusion

In this research, we developed core-shell floating beads for GDDS with porous alginate core and a dense chitosan/xanthan-gum shell layer. The compactness of the shell layer in floating beads was controlled by adjusting the ratios of anionic xanthan gum and cationic chitosan. When the C/X ratio was 4:3 and 4:4, the shell layer would be dense and would cause high resistance of mass transfer under the water penetration. Thus, a low swelling rate and a prolonged release was achieved. The experimental results proved the high biocompatibility of the floating beads, and the anti-bacterial effects of beads were also significant after the release for 4 h. This study proposed the method to modify the properties of shell layer, allowing the control of the swelling and release behaviors of floating beads.

## Data Availability

All data generated or analyzed in this study are included in this published article.

## Abbreviations

GDDS: Gastroretentive drug delivery system
SEM: Scanning electron microscopy
FDDS: Floating drug delivery system
HBS: Hydrodynamically balanced system
MIC: Minimum inhibitory concentration
C/X ratio: Chitosan/xanthan-gum ratio
DMEM: Dulbecco’s modified eagle medium
FBS: Fetal bovine serum
ANOVA: Analysis of variance

## References

[1] Patel SS, Ray S, Thakur RS (2006). Formualtion and evaluation of floating drug delivery system containing clarithromycin for helicobacter pylori. Acta Pol Pharm.

[2] Nayak RMAK, Biswarup D (2010). Gastroretentive drug delivery systems: a review. Asian J Pharm Clin Res.

[3] Arora S, Ali J, Ahuja A, Khar RK, Baboota S (2005). Floating drug delivery systems: a review. AAPS Pharm Sci Tech.

[4] Shah S, Qaqish R, Patel V, Amiji M (1999). Evaluation of the factors influencing stomach-specific delivery of antibacterial agents for helicobacter pylori infection. J Pharm Pharmacol.

[5] Yokel RA, Dickey KM, Goldberg AH (1005). Selective adherence of a sucralfate-tetracycline complex to gastric ulcers: implications for the treatment of *Helicobacter pylori*. Biopharm Drug Dispos.

[6] Umamaheshwari RB, Suman R, Jain NK (2004). Anti *helicobacter pylori* effect of mucoadhesive nanoparticle bearing amoxicillin in experimental gerbils. APPS Pharm Sci Tech.

[7] Kawashima Y, Niwa T, Takeuchi H, Hino T, Ito Y (1991). Preparation of multiple unit hollow microspheres (microballoons) with acrylic resin containing tranilast and their drug release characteristics (*in vitro*) and floating behavior (*in vivo*). J Control Release.

[8] Sato Y, Kawashima Y, Takeuchi H, Yamamoto H (2004). In vitro evaluation of floating and drug releasing behaviors of hollow microspheres (microballoons) prepared by the emulsion solvent diffusion method. Eur J Pharm Biopharm.

[9] Sato Y, Kawashima Y, Takeuchi H, Yamamoto H (2003). Physicochemical properties to determine the buoyancy of hollow microspheres (microballoons) prepared by the emulsion solvent diffusion method. Eur J Pharm Biopharm.

[10] Thanoo BC, Sunny MC, Jayakrishnan A (1993). Oral sustained-release drug delivery systems using polycarb onate microspheres capable of floating on the gastric fluid. J Pharm Pharmacol.

[11] Xu X, Sun M, Zhi F, Hu Y (2006). Floating matrix dosage form for phenoporlamine hydrochloride based on gas forming agent: in vitro and in vivo evaluation in healthy volunteers. Int J Pharm.

[12] Choi B, Park HJ, Hwang S, Park J (2002). Preparation of alginate beads for floating drug delivery system: effects of CO2 gas-forming agents. Int J Pharm.

[13] El-Kamel A, Sokar M, Al Gamal S, Naggar V (2001). Preparation and evaluation of ketoprofen floating oral delivery system. Int J Pharm.

[14] Manca ML, Manconi M, Lai F, Loy G, Matricardi P, Fadda AM (2012). Liposomes coated with chitosan-xanthan gum (chitosomes) as a potential carriers for pulmonary delivery of rifampicin. J Pharm Sci.

[15] Kulkarni N, Wakte P, Naik J (2015). Development of floating chitosan-xanthan beads for oral controlled release of glipizide. Int J Pharm Investig.

[16] Fareez IM, Lim SM, Mishra RK, Ramasamy K (2015). Chitosan coated alginate-xanthan gum bead enhanced pH and thermotelerance of lactobacillus plantarum LAB12. Int J Biol Macromol.

[17] Ferrua MJ, Singh RP (2015). Human gastric simulator (Riddet model), The impact of food bioactives on health.

[18] Al-Dhafri K, Ching CL, Philip K (2019). Phyto-synthesis of silver nanoparticles and its bioactivity response towards nosocomial bacterial pathogens. Biocatal Agric Biotechnol.

[19] Briones AV, Sato T (2010). Encapsulation of glucose oxidase (GOD) in polyelectrolyte complexes of chitosan–carrageenan. React Funct Polym.

[20] Cárdenas A, Argüelles-Monal W, Goycoolea F, Higuera-Ciapara I, Peniche C (2003). Diffusion through membranes of polyelectrolyte complex of chitosan and alginate. Macromol Biosci.

[21] Jin S, Liu M, Chen S, Gao C (2010). A drug-loaded gel based on polyelectrolyte complexes of poly (acrylic acid) with poly (vinylpyrrolidone) and chitosan. Mater Chem Phys.

[22] Singh BN, Kim KH (2000). Floating drug delivery systems: an approach to oral controlled drug delivery via gastric retention. J Control Release.

[23] Mi FL, Shyu SS, Wong TB, Jang SF, Lee ST, Lu KT (1999). Chitosan polyelectrolyte complexation for the preparation of gel beads and controlled release of anticancer drug. II. Effect of pH- dependent ionic crosslinking or interpolymer complex using tripolyphosphate or polyphosphate as reagent. J Appl Polym Sci.

[24] Mi FL, Chen CT, Tseng YC, Kuan CY, Shyu SS (1997). Iron (III)- carboxymethylchitin microsphere for the pH-sensitive release of 6- mercaptopurine. J Control Release.

[25] Shu X, Zhu KJ (2000). A. novel, approach to prepare tripolyphosphate/ chitosan complex beads for controlled release drug delivery. Int J Pharm.

